# The ArathEULS3 Lectin Ends up in Stress Granules and Can Follow an Unconventional Route for Secretion

**DOI:** 10.3390/ijms21051659

**Published:** 2020-02-28

**Authors:** Malgorzata Dubiel, Tibo De Coninck, Vinicius Jose Silva Osterne, Isabel Verbeke, Daniël Van Damme, Guy Smagghe, Els J. M. Van Damme

**Affiliations:** 1 Laboratory of Biochemistry and Glycobiology, Department of Molecular Biotechnology, Ghent University, Coupure Links 653, 9000 Ghent, Belgium; Malgorzata.Dubiel@UGent.be (M.D.); Tibo.DeConinck@UGent.be (T.D.C.); VinnyOsterne@Alu.Ufc.br (V.J.S.O.); Isabel.Verbeke@UGent.be (I.V.); 2 Laboratory of Agrozoology, Department of Plants and Crops, Ghent University, 9000 Ghent, Belgium; Guy.Smagghe@UGent.be; 3 Laboratório de Moléculas Biologicamente Ativas, Universidade Federal do Ceará, José Aurelio Camara, S/N, 61440-970, Fortaleza 60440-970, Brazil; 4 Department of Plant Biotechnology and Bioinformatics, Ghent University, Technologiepark 71, 9052 Ghent, Belgium; Daniel.VanDamme@psb.vib-ugent.be; 5 VIB Center for Plant Systems Biology, Technologiepark 71, 9052 Ghent, Belgium; 6 Center for Advanced Light Microscopy, Ghent University, 9000 Ghent, Belgium

**Keywords:** *Arabidopsis*, ArathEULS3, intrinsically disordered regions, plant lectin, stress granules, unconventional protein secretion

## Abstract

Stress granules are cytoplasmic compartments, which serve as mRNA storage units during stress, therefore regulating translation. The *Arabidopsis thaliana* lectin *ArathEULS3* has been widely described as a stress inducible gene. This study aimed to examine in detail the localization of ArathEULS3 lectin in normal and stressed cells. Colocalization experiments revealed that the nucleo-cytoplasmic lectin ArathEULS3 relocates to stress granules after stress. The ArathEULS3 sequence encodes a protein with a EUL lectin domain and an N-terminal domain with unknown structure and function. Bioinformatics analyses showed that the N-terminal domain sequence contains intrinsically disordered regions and likely does not exhibit a stable protein fold. Plasmolysis experiments indicated that ArathEULS3 also localizes to the apoplast, suggesting that this protein might follow an unconventional route for secretion. As part of our efforts we also investigated the interactome of ArathEULS3 and identified several putative interaction partners important for the protein translation process.

## 1. Introduction

In plants, non-membrane bound structures containing high amounts of proteins and RNAs (mRNPs—messenger ribonucleoproteins) are represented by nuclear Cajal bodies, paraspeckles and nucleoli, as well as cytoplasmic protein bodies (PBs) and stress granules [1]. Stress granules are cytoplasmic aggregates composed of polyadenylated mRNA, 40S ribosomal subunits, poly(A)-binding proteins, and eukaryotic initiation factors (eIFs), like eIF3, eIF4e, eIF4A, as well as RNA helicases, RNA binding proteins, such as PABP, RAP47B, UBP1, and regulators of mRNA decay and translation [2,3]. Stress granules have been reported in all eukaryotes: animals, yeasts, and plants [4,5,6]. Recent studies suggest that stress granules become important when the plant is exposed to abiotic stress conditions. The formation of stress granules in plants is induced in high salinity conditions, osmotic stress, drought, hypoxia, and after heat stress [2,7,8]. The stress granules store mRNAs, which are not being translated at the moment and are stabilized or undergo degradation [3,9]. When the stress factor dissipates, the stress granules disassemble and the mRNA can be translated [1]. The PBs on the other hand, serve as mRNA degradation machinery and contain inactive mRNAs and proteins involved in inhibiting translation and mRNA degradation [2]. The PBs are present in non-stressed cells and their number can increase under stress conditions. In yeast and mammalian cells, it was shown that PBs and stress granules can overlap [1]. Until recently, most of the research investigating stress granules in plants was based on the protein homologs present in mammalian stress granules. The first report on plant stress granules proteomics was published only a few months ago, when Kosmacz et al., (2019) managed to isolate the stress granules by combining differential centrifugation with affinity purification followed by identification of the stress granules proteome [10]. Next to the predicted and known stress granules proteins, like TSN1 and TSN2 [2], a significant amount of proteins, important for plant stress responses, were identified such as cyclin-dependent kinase A, elongation initiation factors, chaperones, and RNA binding proteins as well as nucleotides, amino acids, and phospholipids. Ras-GTPase-activating protein SH3-domain-binding (AtG3BP1), an RNA binding protein, was identified as a negative regulator of defense responses. *Arabidopsis g3bp1* mutants were more resistant to *Pseudomonas syringae* pv. tomato by restricting stomatal pathogen entry [11].

Lectins are proteins found in all living organisms. They reversibly bind to carbohydrates or glycan structures, without changing their properties. Lectins are composed of one or more lectin domains, sometimes combined with other protein domains [12]. Most of the classical, well-researched lectins have a storage function and/or defense-related role and localize to the plant vacuole or are secreted [13]. The family of *Euonymus europaeus* related lectins (EUL) represents a group of stress-inducible lectins that show homology to the *Euonymus europaeus* agglutinin (EEA) [14]. The genome of the model plant *A. thaliana* contains only one *EUL* gene, further referred to as *ArathEULS3*. Induced expression of *ArathEULS3* was reported after hormone treatments (abscisic acid, ABA), and after abiotic (drought, salt, and osmotic stress) and biotic (*Pseudomonas syringae*) stress [15]. The *ArathEULS3* lectin sequence is synthesized with a long N-terminal domain of unknown function. In line with the fact that the *ArathEULS3* sequence does not contain a signal peptide, the lectin was shown to localize to the nucleo-cytoplasmic compartment of plant cells [16]. 

In general, protein secretion is a conserved process in all eukaryotes, which relies on the presence of an N-terminal signal peptide sequence. Secretion directs proteins through the endoplasmic reticulum, Golgi, and Trans-Golgi network, via vesicular transport until they fuse with the plasma membrane and release their cargo to the extracellular space. However, growing evidence from proteomic studies indicates that over 50% of all secreted proteins lack a signal peptide sequence suggesting a possibility for unconventional secretion of leaderless proteins (LPs) [17]. In plants, unconventional protein secretion relies on the presence of vesicles, which contain cytoplasmic proteins inside a double-membrane vesicle or attached to the vesicular membrane. Those vesicles are secreted through fusion with the plasma membrane, releasing extracellular vesicles (EVs) containing LPs into the apoplast. EVs in plants were first observed in carrot cells in the 1960s by Halperin and Jensen (1967) [18]. To date, three sources of EVs in plants have been confirmed including exosomes arising from the multivesicular bodies (MVBs, also called “late endosomes” or “pre-vacuolar compartment”), the vacuole, and the exocyst-positive organelle (EXPO). EXPOs are recognized by the presence of the Exo70 protein and are not affected by brefeldin A, Concanamycin A, and Wortmannin treatments [19]. When secreted, single membrane vesicles burst open due to the change of osmolarity and release their content. S-adenosylmethionine synthetase 2 (SAMS2), a lignin biosynthetic enzyme, and glycosyltransferases important for glycosylation of arabinogalactan proteins such as AtGALT14A, AtGALT29A, and AtGALT31A are examples of proteins secreted through EXPOs [20]. EVs can also arise from vacuole-plasma membrane fusion as part of programmed cell death [21]. Vacuoles contain defense proteins and hydrolytic enzymes, which after secretion are likely to kill the pathogens such as *Pseudomonas syringae* or tobacco mosaic virus [22,23]. MVBs, next to cytoplasmic proteins also carry small RNAs and serve as another alternative route for secretion of LPs [24]. The amount of EVs was enhanced after infection with powdery mildew fungus in barley [25], *Pseudomonas syringae* in *Arabidopsis* plants [26,27], *Botrytis cinerea* in *Arabidopsis* cells or Turnip mosaic virus infection of tobacco leaves [28]. Recently, Rutter and Innes (2017) examined the proteome of EVs from *Arabidopsis*, indicating the presence of many stress responsive proteins, including lectins, characteristic for biotic and abiotic stresses [27]. EVs isolated from extracellular fluids of sunflower seedlings contained cell wall remodeling enzymes and defense proteins, suggesting the role of EVs in plant immune responses [29].

Here we present a thorough study of the localization ArathEULS3, both under normal conditions as well as after heat stress using colocalization analyses in *Nicotiana benthamiana* of enhanced Green Fluorescent Protein (EGFP)-tagged ArathEULS3 combined with markers visualizing peroxisomes, stress bodies, and autophagosomes. Detailed analysis of the *ArathEULS3* sequence and its folding points to the presence of intrinsically disordered regions (IDRs) in the N-terminal domain. Furthermore, the identification of several putative interaction partners for ArathEULS3 using the pull-down analysis with a full length, recombinantly produced ArathEULS3 protein provides insight into the physiological function of ArathEULS3 pointing towards its possible role in regulation of translation under stress.

## 2. Results

### 2.1. Nucleo-cytoplasmic ArathEULS3 Relocates to Stress Granules 

**Figure 1:**
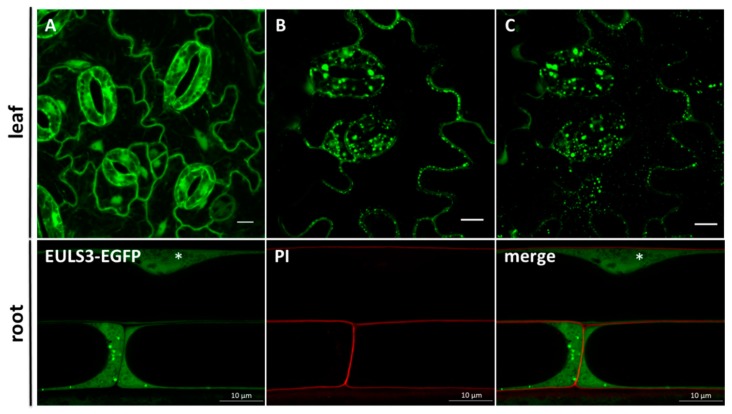
Localization of ArathEULS3-EGFP in *Arabidopsis thaliana*. (**A**–**C**) Confocal images representing unstressed *Arabidopsis* epidermal leaf cells. Panels A and C show a compilation of fluorescent images acquired along the z-axis. Panel B represents single slice optical sections. The lower panel represents ArathEULS3-EGFP localization in *A. thaliana* roots. The asterisks indicate the position of the nucleus. Confocal images are single slice optical sections. PI (propidium iodide) and EGFP channels are combined in the overlay picture (merge). Scale bars represent 10 µm.

The subcellular localization of ArathEULS3 was studied in *A. thaliana* lines expressing 35S::ArathEULS3-EGFP. Fluorescence was observed in the nucleus as well as in the cytosol of leaf and root cells of plants grown under normal growth conditions (Figure 1A). In addition, punctate structures throughout the cytoplasm were observed in 90% of non-stressed leaf and root cells (Figure 1). *A. thaliana* plants stably expressing free EGFP grown under identical conditions were used as the control, and showed EGFP fluorescence in the nucleus and the cytoplasm, but did not show any punctate structures (Supplementary Figure S1).

**Figure 2:**
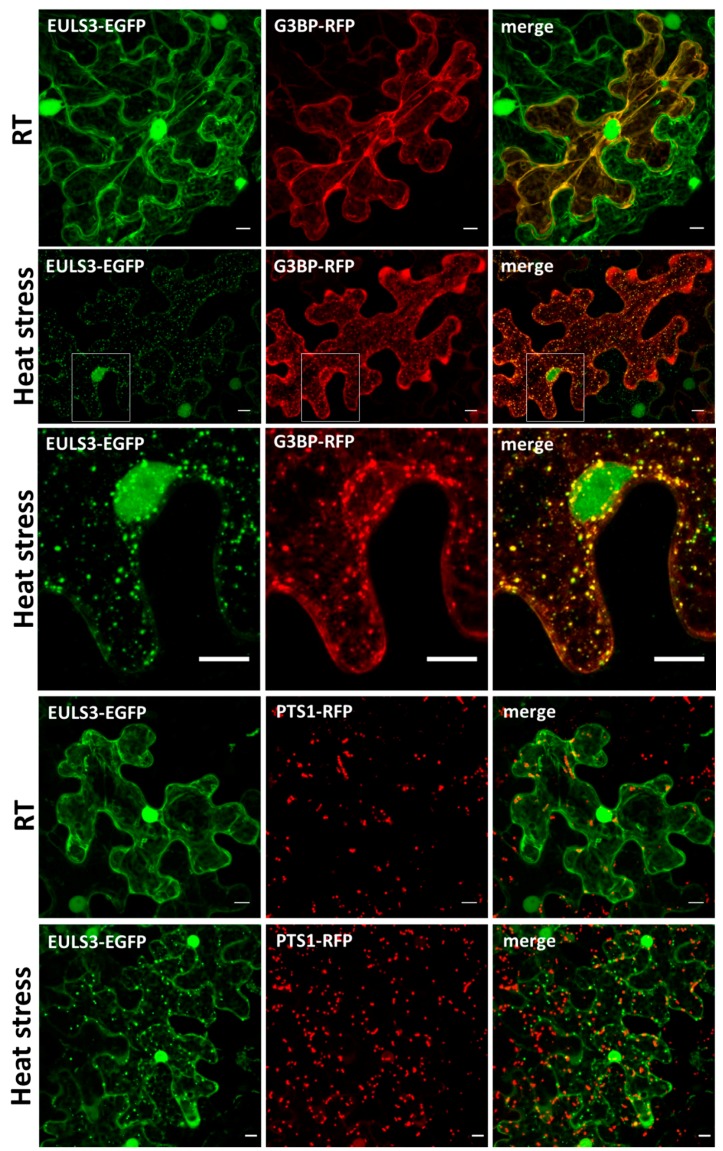
Colocalization of ArathEULS3-EGFP with the G3BP-RFP stress granules marker and the PTS1-RFP peroxisome marker. Confocal images represent epidermal cells of *Nicotiana* *benthamiana* leaves after transient coexpression of ArathEULS3-EGFP with G3BP-RFP at room temperature (RT) and after 45 min of heat treatment at 37 °C. All images show a projection of fluorescent images acquired along the z-axis. Scale bars represent 10 µm.

**Figure 3:**
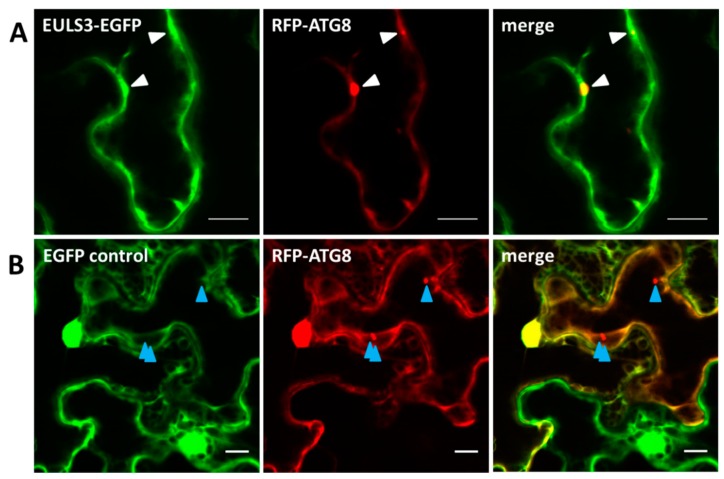
Colocalization of ArathEULS3-EGFP with RFP-ATG8 autophagy marker. (**A**) Transient coexpression of ArathEULS3-EGFP with RFP-ATG8 in *N. benthamiana* leaves at RT. (**B**) Control experiment showing transient coexpression of free-EGFP with RFP-ATG8 in *N. benthamiana* leaves. White triangles point to punctate structures where the GFP and RFP signals overlap and blue triangles indicate absence of colocalization. Scale bars represent 10 µm.

To investigate the nature of the punctate structures, colocalization experiments with several RFP-tagged organelle markers including PTS1 (peroxisome), G3BP (stress granules), and ATG8 (autophagy) were performed by transient transformation of *N. benthamiana* leaves. In tobacco plants grown under non-stressed conditions the G3BP protein localized to the cytoplasm. However, soon after plants were subjected to heat treatment G3BP clearly located to stress granules. Double transformations with ArathEULS3 and G3BP revealed colocalization between the G3BP-RFP and ArathEULS3-EGFP fluorescence in the cytoplasm of non-stressed plants (Figure 2). A prominent colocalization between ArathEULS3-EGFP and G3BP-RFP was observed after heat stress (Figure 2), but was absent when free EGFP was analyzed in combination with G3BP-RFP (Supplementary Figure S2). After heat stress, punctate structures for ArathEULS3-EGFP were also present in the nucleus (Supplementary Figure S3). These nuclear dots of ArathEULS3-EGFP did however not colocalize with G3BP-RFP, as G3BP-RFP never localized to the nucleus. Coexpression analysis of ArathEULS3-EGFP and the peroxisome marker PTS1-RFP revealed no significant colocalization (Figure 2). Additionally the colocalization of ArathEULS3-EGFP and the RFP-tagged autophagy marker ATG8 was examined. Colocalization between ArathEULS3 and RFP-ATG8 was examined as both autophagosomes and ArathEULS3 positive bodies localize to the cytoplasm. However, no convincing evidence for the presence of ArathEULS3-EGFP on the autophagy bodies was obtained. Similarly, in *N. benthamiana* leaves transiently expressing the free EGFP protein as a control, no colocalization between ArathEULS3-EGFP and the ATG8 marker was observed (Figure 3B). Together these data suggest that ArathEULS3 localizes to the cytoplasm, nucleus as well as the stress granules, but likely not to autophagosome.

### 2.2. ArathEULS3 Is a Non-conventionally Secreted Protein

**Figure 4:**
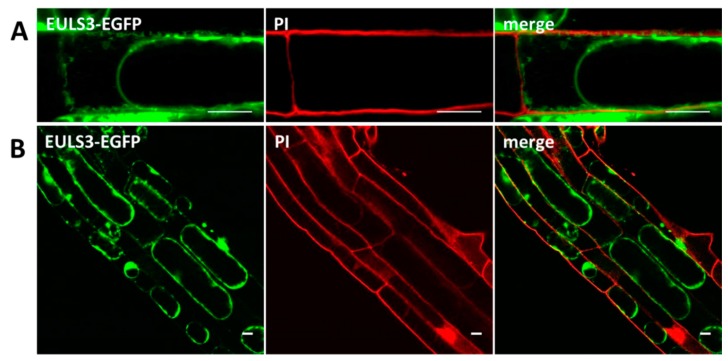
ArathEULS3-EGFP localization in the apoplast. (**A**,**B**) Confocal images of plasmolyzed root cells showing fluorescence of ArathEULS3-EGFP in the apoplast. Plasmolysis was performed using 0.8 M sorbitol. Confocal images are single slice optical sections. PI and EGFP channels are combined in overlay pictures (merge). Scale bars represent 10 µm.

Predictions using SignalP indicated that the *ArathEULS3* sequence does not contain a signal peptide sequence. Therefore ArathEULS3 is likely to be synthesized on the free ribosomes residing in the cytoplasm. Predictions for ArathEULS3 localization in the cell using different software packages [30] yielded contradicting results. In line with the localization patterns observed for the lectin-EGFP fusion constructs, PSORT and Cello predicted localization in the nucleus. In contrast, the results suggested by SubLoc indicated an extracellular location (84% expected accuracy). Similarly LocTree3 [31] reported ArathEULS3 as a secreted protein (95% expected accuracy). According to the SecretomeP software [32] ArathEULS3 is predicted to act as a non-classical secretory protein with a significant score. Staining of *A. thaliana* root cells stably expressing ArathEULS3-EGFP using PI allowed us to differentiate between the cytoplasm and the plasma membrane. Fluorescence intensity plots of PI and EGFP cross sections spanning the cell borders of neighboring cells did not show the presence of the lectin in the cell wall (Supplementary Figure S4). To evaluate whether ArathEULS3 exits the cell, a plasmolysis assay was performed. Plasmolysis of root cells overexpressing ArathEULS3-EGFP using 0.8 M sorbitol revealed a significant fluorescence signal in the apoplast (Figure 4), which was not observed in the control experiments with EGFP (Supplementary Figure S5). Similarly fluorescence was observed after plasmolysis of *A. thaliana* root cells with stable expression of ArathEULS3-RFP (Supplementary Figure S6), suggesting that ArathEULS3 is also present beyond the plasma membrane.

### 2.3. ArathEULS3 Sequence Analysis

**Figure 5:**
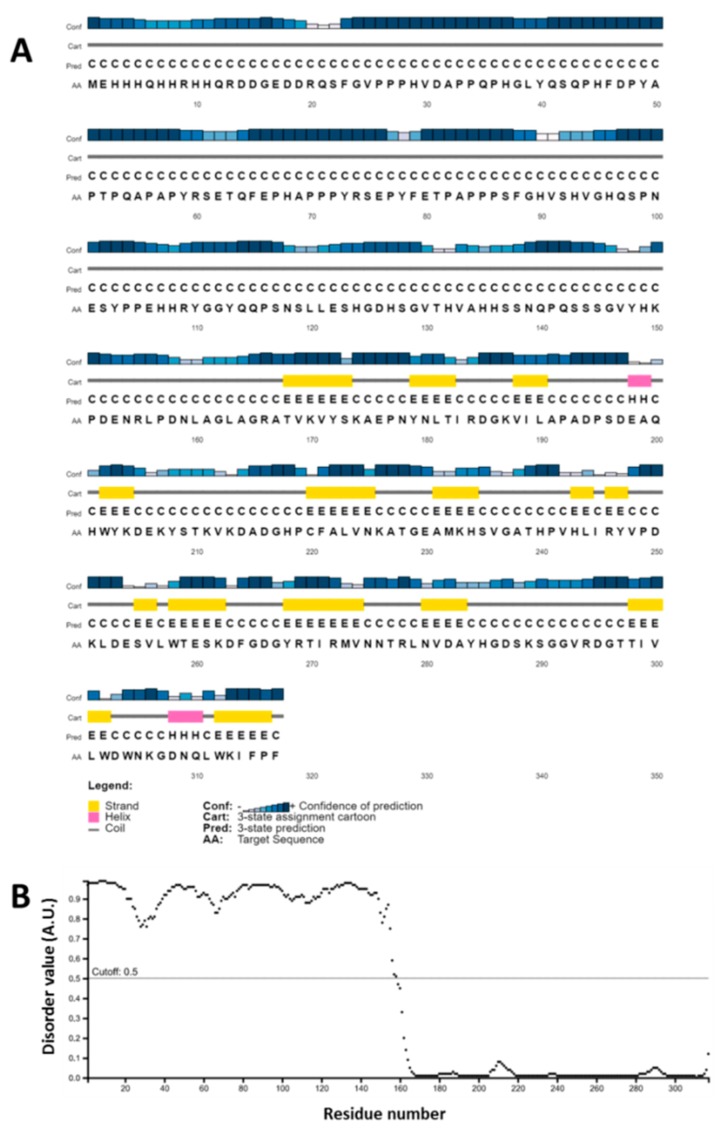
Amino acid sequence analysis of ArathEULS3. (**A**) Secondary structure prediction by PsiPred 4.0. The colors represent protein secondary structure elements (yellow for β-strands, pink for α helix and grey for coil structures). (**B**) Disorder plot by DISOPRED3.

The ArathEULS3 sequence consists of an N-terminal domain (163 amino acids) followed by the EUL domain (154 amino acids), encoding a protein of approximately 36 kDa. Secondary structure predictions suggested an unexpected pattern for the N-terminal domain that appears to be formed by coiled structures and loops, while the lectin domain mainly revealed the presence of β-strands (Figure 5A). This result corroborated with the disorder plot that indicated a very high disorder from residue 1 to residue 160 followed by a low disorder in the second domain. The large prevalence of loops in the first half of *ArathEULS3* sequence can be characterized as IDRs (Figure 5B) that consist of polypeptide segments that likely do not fold into a defined three-dimensional structure, but are functional [33,34]. Moreover, a search by a conserved domain in NCBI suggested that the N-terminal domain of ArathEULS3 had the conserved domain of a DNA binding protein of the topoisomerase II family, which has also been shown to contain the IDRs [35]. The lectin domain sequence (residues 164-317) not surprisingly, indicated the presence of a Ricin-type beta-trefoil lectin domain, which is expected for proteins presenting EUL domains. No reliable model for ArathEULS3 could be generated because of the low sequence identity between the template and the N-terminal region of ArathEULS3.

### 2.4. ArathEULS3 Protein Purification 

**Figure 6:**
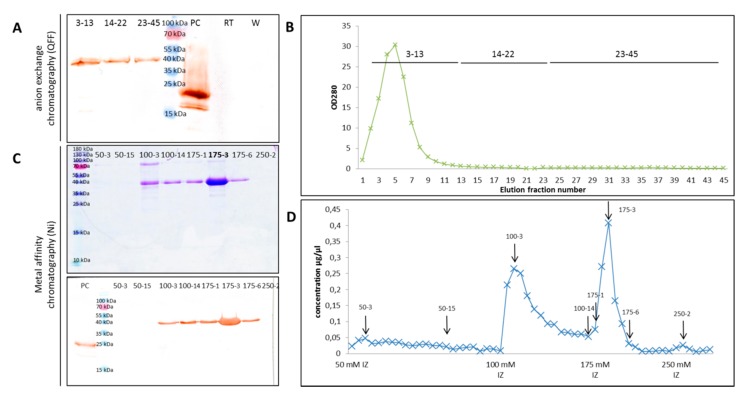
ArathEULS3 protein purification. (**A**) Western blot analysis of proteins eluted from anion exchange chromatography on Q Fast Flow (QFF). RT—run through, PC—HIS-tagged positive control protein (lectin domain of OsEULS2, LOC_Os07g48500.1, MW: 22.8 kDa), and W—wash. (**B**) Graph illustrating the elution fractions from the QFF column. Samples indicated are analyzed in panel A. (**C**) SDS-PAGE and Western blot analysis of proteins eluted from metal affinity chromatography (Ni-NTA agarose beads). (**D**) Graph illustrating the elution fractions from the Ni-NTA column. Arrows indicate the samples analyzed in panel C.

Recombinant Myc- and His-tagged ArathEULS3 protein was successfully produced in *Pichia pastoris* strain X-33 and purified using a combination of anion exchange and metal affinity chromatography (Figure 6). After the first chromatography step samples with similar OD280 values were pooled and subjected to Western blot analysis. ArathEULS3 was detected in all the elution samples, but was absent from the run through and the wash fractions. After affinity purification using Ni-NTA matrix, the protein was present in all elution fractions except for samples eluted with 50 mM and 250 mM imidazole. Since some impurities were present in the protein fractions eluted with 100 mM imidazole only the fractions eluted with 175 mM imidazole were used for further experiments.

### 2.5. Pull-Down Analysis Reveals Several Novel Candidate Interactors for ArathEULS3

**Figure 7:**
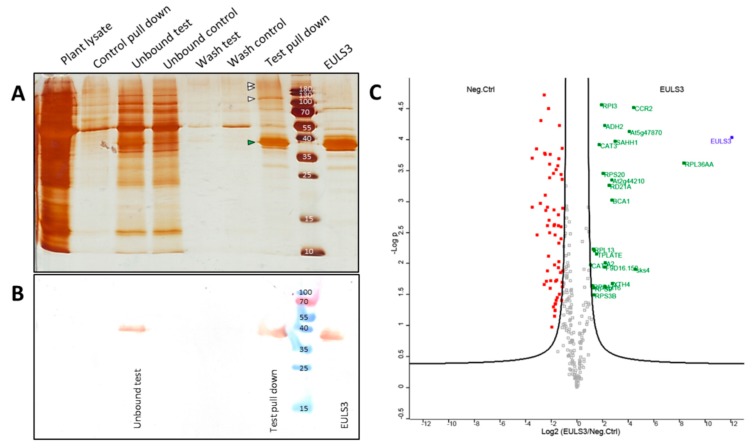
A pull down analysis of ArathEULS3. (**A**) Silver stained SDS-PAGE illustrating the protein patterns in the following order: crude plant lysate (prey), control pull down (Myc-trap^®^ beads incubated with plant lysate), proteins that did not bind to the beads for test and control pull-down, washes of Myc-trap^®^ beads, test pull-down (proteins eluted from the Myc-trap^®^ beads incubated with ArathEULS3 lectin), and purified recombinant ArathEULS3. (**B**) Western blot analysis using the anti-ArathEULS3 antibody. (**C**) Volcano plot of proteins identified by mass spectrometry (*N* = 3). The ArathEULS3 protein is indicated in blue, 31 significantly enriched proteins are shown in green.

To get more insight in the functional role of ArathEULS3, we searched for proteins interacting with the lectin. Since ArathEULS3 is known as an ABA responsive protein a pull-down experiment was performed using recombinant ArathEULS3 as a bait and plant lysates from a 2-week-old wild type, *A. thaliana* plants treated with 100 µM ABA as the prey. Analysis of the proteins captured by the Myc-trap^®^ beads loaded with recombinant ArathEULS3 compared to the beads in the control sample revealed some unique polypeptide bands between 100 and 130 kDa and above 180 kDa (Figure 7A), which could indicate possible interaction partners of ArathEULS3. The fact that ArathEULS3 was detected on the Western blot in the unbound fraction of the pull-down samples, but not in the negative control, indicates that the beads were saturated with the ArathEULS3 protein (Figure 7B). ArathEULS3 was not detected by Western blot analysis in the plant lysate nor in any of the washes from the beads. 

Three independent experiments were performed and putative interactors were analyzed by LC–MS/MS. Out of 10,459 identified peptides, 424 proteins were identified (Supplementary Table S1). In total 262 proteins were reliably quantified (Supplementary Table S2). Comparative analysis between lectin samples and the control samples allowed to exclude proteins bound specifically to the beads, resulting in 23 significantly enriched proteins, that were either uniquely present or had a significantly higher abundance in the bait samples in comparison to the negative control samples (Table 1, Figure 7C). Mass spectrometry identified multiple proteins important for the translation process, such as elongation factors. In addition, some stress-related proteins (catalase-2 and catalase-3) were retrieved with high scores. Considering the carbohydrate-binding activity of the ArathEULS3 lectin domain, all secreted proteins identified as putative interaction partners were analyzed for the presence of glycosylation sites. No glycosylation sites were predicted with significant scores (above 0.5) for the Xyloglucan endotransglucosylase/hydrolase sequence. Several glycosylation sites were found both for the Pectin esterase-like protein SKU5 and the F4I1.2 uncharacterized protein sequences.

## 3. Discussion

Stress granules are cytoplasmic compartments responsible for translational repression through mRNA storage during stress. They consist mainly of RNA-binding proteins, translation initiation factors, 40S ribosomal subunits, and heat shock proteins [2]. In this study, we provided evidence suggesting that the lectin ArathEULS3 lectin was relocated from the cytoplasm to the stress granules upon heat stress. Previously it was shown that ArathEULS3 is located in the nucleus and the cytoplasm of tobacco BY-2 cells [16]. The nucleocytoplasmic localization was confirmed using transgenic lines of *Arabidopsis* overexpressing ArathEULS3-EGFP. Next to the nucleocytoplasmic localization, the presence of fluorescent cytoplasmic compartments was observed when analyzing the localization of ArathEULS3-EGFP in most *Arabidopsis* leaves of plants grown in unstressed conditions. These fluorescent dots were absent in *N. benthamiana* leaves transiently transformed using ArathEULS3-EGFP. The cytoplasmic compartments containing the lectin only became visible in *N. benthamiana* leaves after application of heat stress. This difference in response between *Arabidopsis* and tobacco could be explained by the young age of *Arabidopsis* plants (9 day-old) that were used for the preparation of microscopic slides inducing stress responses, compared to the 3-4 week-old *N. benthamiana* plants, which are more robust at this stage. Colocalization studies with the stress granule marker, G3BP-RFP in *N. benthamiana* transiently expressing ArathEULS3 suggested that the lectin is relocated to stress granules after the plant has been subjected to certain stresses. Similar results were reported for other plant proteins such as the polyadenylated (poly(A)+) mRNA binding protein PAB-2 [7] and the VASCULAR PLANT ONE-ZINC FINGER (VOZ)1/and VOZ2 [36], which in normal conditions localize to the cytoplasm and the nucleus, and are translocated to stress granules upon stress. However, despite the fact that ArathEULS3 transcript levels are known to be upregulated in response to drought stress the ArathEULS3 protein was not identified in the proteome of heat/dark treated plants [10] nor was it identified when examining the changes in RNA-binding proteome under drought stress conditions [9,37]. This might be explained by the fact that the proteome of the stress granules might change depending on the nature and severity of the stress conditions as well as the developmental stage of the plant [1]. Additionally the stress granules proteome analysis revealed elongation factors or chaperons, the presence of stress-related proteins such as receptors, kinases, and transcriptional regulators next to the predicted stress granule components like RNA-binding proteins [10]. Considering the stress-responsive nature of ArathEULS3, more proteomic analyses performed under different stress conditions will help to confirm the presence of ArathEULS3 protein in stress granules. 

In search for ArathEULS3 interaction partners in plants subjected to ABA stress, a pull-down experiment was performed. Mass spectrometry identified several proteins responsible for the translation process, such as ribosomal proteins and elongation factors. Interestingly, 30% of all proteins identified as putative interaction partners for ArathEULS3 were also identified during the analysis of the mRNA-protein interactome performed by Reichel et al. (2016): Glycine-rich RNA-binding protein 7 (At2g21660), Adenosyl homocysteinase (At4g13940), Xyloglucan endotransglucosylase (At2g06850), Elongation factor 1-alpha 1/2/3 (At1g07930, At1g07920, At1g07940), S-(hydroxymethyl) glutathione dehydrogenase (At5g43940), Catalase-3 (AT1G20620), 40S ribosomal protein S3-2 (At3g53870), and 50S ribosomal protein L21 (AT1G35680) [38]. A double-domain EUL from rice (*Oryza sativa*)—a close homologue of ArathEULS3—was also identified as an RNA-binding protein in an analysis using single stranded DNA affinity column chromatography [39]. One of the proteins, which was identified in our analysis as a putative interactor for ArathEULS3, the Glycine-rich RNA-binding protein, was presented as an RNA-binding protein in the rice seeds. There is a possibility, that the rice EUL protein was identified among the single stranded DNA-interacting proteins not through direct interaction with the DNA, but through indirect interaction through the Glycine-rich RNA-binding protein. Interestingly, ANGUSTIFOLIA, which localizes to stress granules and regulates stress granules formation, was shown to interact also with the Glycine-rich RNA-binding protein 7. The latter protein might therefore be casual to the common localization of ANGUSTIFOLIA and ArathEULS3 in the stress granules [8]. All this evidence suggests that, even if ArathEULS3 does not bind directly to nucleic acids, it could be a part of a larger protein complex together with the Glycine-rich RNA-binding protein, which regulates transcription processes. 

Analysis of the ArathEULS3 protein sequence revealed that the N-terminal domain (residues 1 to 163) could be classified as an IDR while the residues from 164 to 317 correspond to the lectin domain, which folds into a EUL domain. IDRs are regions consisting of polypeptide segments that likely do not fold into a defined three-dimensional structure, but are nonetheless functional [33,34]. It is known that a large majority of eukaryotic proteins (25%-50%) have a modular organization with combinations of structured and disordered regions, which also appears to be the case for ArathEULS3 [40]. A common function for the IDRs is binding to other molecules, with nucleic acids being a prevalent ligand, this information corroborates with the functional prediction using the Conserved Domain Database from NCBI and FFPred 3 that pointed towards nucleic-acid binding activity for the N-terminal domain of ArathEULS3. The clear domain separation in ArathEULS3 indicates that the possible nucleic-acid binding and the carbohydrate-binding activities take place in separate protein domains. Altogether, these data, the nuclear localization and the localization in stress granules after stress, suggest that ArathEULS3 might bind to nucleic acids. Interestingly, Protter and Parker (2016) suggested that many proteins found in stress granules contain IDRs and some of them, like the human TIA-1 protein, promote stress granule formation [1]. Therefore, the IDRs are proposed to play a role in stress granule assembly and stabilization, but the evidence showing the influence of IDRs on stress granule formation is still limited.

Furthermore, when analyzing the localization of ArathEULS3 after heat stress, we observed the presence of punctate structures inside the nucleus. Since stress granules are cytoplasmic compartments, it seems that ArathEULS3 can also bind to other structures in the nucleus. However, we were not able to identify these spots inside the nucleus. It is known that IDRs are present in proteins involved in signaling and transcriptional regulation [41], and eukaryotic transcription factors have a large fraction of IDRs responsible for recognition and interaction with other coactivators/co-repressors and other transcription factors [42,43]. Considering that the nucleus is the main site in the cell for DNA replication and transcription, it is likely that ArathEULS3 might bind to complexes composed of proteins and nucleic acids within the nucleus.

In eukaryotic cells, the secretion of proteins is a conserved process. It requires proteins to be synthesized with an N-terminal signal peptide sequence, which helps to direct the protein via the vesicular transport from the endoplasmic reticulum, Golgi, and Trans-Golgi network, to eventually fuse with the plasma membrane, and release the proteins to the extracellular space. Next to the fact that ArathEULS3 is transported to the stress granules upon stress, we have also shown that a small amount of the protein localizes to the apoplast of plasmolyzed cells. ArathEULS3 is a nucleo-cytoplasmic protein, which does not contain the N-terminal signal sequence [16], therefore it must follow an unconventional pathway for secretion. All described routes for unconventional secretion in plants involve a type of double membrane-bound structures (EXPOs, MVBs, or vacuole) [17]. In our research, no additional vesicular structures were observed during microscopy. In mammalian cells and yeast, several examples of LPs were described to be secreted through plasma membrane pore formation or by ABC transporters anchored in the plasma membrane, such as, e.g., described for interleukin (IL)-1β, fibroblast growth factors, and the HIV transactivator of transcription [44]. In plants, direct translocation across the plasma membrane was not discovered yet. Similar to ArathEULS3, the secretion of hygromycin phosphatase and mannitol dehydrogenase was not mediated by any vesicular structures [45,46]. Interestingly, the mammalian Y-box protein YB-1 assembly in stress granules was recently associated with an enhancement of its unconventional secretion [47]. The YB-1 protein is involved in many fundamental biological processes and its localization highly depends on the conditions. In non-stress conditions YB-1 is localized to the cytoplasm, it is a component of mRNPs and after DNA damage it is transported to the nucleus where it helps DNA repair. Under stress conditions YB-1 is important for stress granules formation, and can be secreted. When searching for proteins involved in pathogen-associated molecular pattern-induced changes in plasma membrane compartmentalization, Keinath and coworkers identified ArathEULS3 in a detergent-insoluble plasma membrane fraction [48]. Gilbert and Schulze (2019) identified ArathEULS3 peptides in Arabidopsis root membranes [49]. Additionally, peptides derived from ArathEULS3 were found in the cell wall proteome of *A. thaliana* rosettes and mature stems [50,51]. Moreover, the experiment investigating the cell wall proteome of two *Arabidopsis* ecotypes Columbia (Col) and Shahdara (Sha) grown at 22 °C and at 15 °C, respectively, revealed an increase in ArathEULS3 peptides in both ecotypes at a lower temperature [52]. All these data can serve as additional proof to show that ArathEULS3 is secreted to the extracellular space under stress conditions. 

The ArathEULS3 protein was previously proven to be a functional lectin that interacts with galactosylated glycans with and without core fucosylation [16]. Two out of three secreted putative ArathEULS3 interaction partners revealed putative glycosylation sites after in silico analysis (Pectin esterase-like protein SKU5 similar 4 (SKU5) and F4I1.2 uncharacterized protein). However, according to proteomics analyses performed by Zielinska et al. (2012) only the Pectin esterase-like protein SKU5 similar 4 was detected as a glycosylated protein [53]. Together with the evidence for ArathEULS3 secretion, these data suggest that there is a possibility for ArathEULS3 and SKU5 to interact in the extracellular space, nevertheless additional experimental evidence is necessary to confirm this interaction.

In summary, in this study we present several novel findings concerning the localization of ArathEULS3. We show that upon stress ArathEULS3 localizes to the stress granules and likely interacts with proteins involved in translation. Moreover, the recognition of the IDRs in the N-terminal domain of ArathEULS3 helps further explaining the possible interaction with DNA or RNA or other proteins being a part of protein complexes capable of binding nucleic acids. Additionally, next to the available proteomics data, we delivered experimental proof for the unconventional secretion of the nucleo-cytoplasmic lectin ArathEUL3.

## 4. Materials and Methods 

### 4.1. Plant Material and Growth Conditions

Wild type *A. thaliana* seeds, ecotype Columbia, were kindly supplied by Prof. Dr. Richard Strasser (Department of Applied Genetics and Cell Biology, University of Natural Resources and Life Sciences, Vienna, Austria). Seeds were surface-sterilized in 70% (*v/v*) ethanol for 2 min, followed by 8 min in 5% NaClO (Sigma-Aldrich, St. Louis, MI, USA). Afterwards, the seeds were rinsed eight times with sterile distilled water. The sterilized *Arabidopsis* seeds were sown in vitro on solid ½ Murashige and Skoog (MS) medium (2.154 g/L MS basal salt (Duchefa Biocheme, Haarlem, Netherlands), 10 g/L sucrose (Duchefa Biocheme), 0.1 g/L myo-inositol (Duchefa Biocheme), 0.5 g/L MES—2-(N-morpholino)ethanesulfonic acid (Roth, Karlsruhe, Germany), and 8 g/L plant tissue culture agar (Duchefa Biocheme), pH 5.7). After stratification for 3 days at 4 °C in the dark the plates were transferred to a growth chamber at 21 °C with a 16/8 h light/dark photoperiod for 3-4 days for root assays and 9 days for leaf assays. *N. benthamiana* seeds were kindly provided by Dr. Verne A. Sisson (Oxford Tobacco Research station, Oxford, NC, USA). *N. benthamiana* plants were sown in commercial soil in pots and kept at 28 °C under normal light conditions with a 16/8 h light/dark photoperiod.

### 4.2. Construction of Expression Vectors and Plant Transformation 

A plasmid harboring the full length cDNA, coding for ArathEULS3 (At2g39050) was ordered from the Experimental Plant Division group within the Department of Biological Systems of the BioResource Center of the RIKEN Tsukuba Institute (Ibaraki, Japan) [54,55]. Fusion of ArathEULS3 to EGFP or RFP was achieved by means of the Gateway™ technology (Invitrogen, Carlsbad, CA, USA). The ArathEULS3 sequence was cloned into pK7FWG2 and pK7RWG2 vectors for C-terminal EGFP and RFP fusion, respectively. The cloning procedure of ArathEULS3 with C- and N-terminal EGFP fusion protein is described in detail by Van Hove, et al. (2011) [16]. *A. thaliana* plants ecotype Columbia were stably transformed with the expression vectors for the 35-S promoter driven expression of ArathEULS3 with C-terminal EGFP and RFP tag via Agrobacterium-mediated floral dip method [56]. Plant selection was done by planting seeds on ½ MS medium supplemented with 75 μg/mL kanamycin. For microscopy analyses the root cell walls of 3–4 day-old seedlings were stained with PI, 10 µg/mL (Sigma-Aldrich) for 15 s and on microscopy slides in water. A peroxisome organelle marker PTS1-RFP was available from the Nottingham *Arabidopsis* Stock Centre (NASC), stock number CD3-983. Plasmids containing the pUBQ10::3xHA-TagRFP-AtG8a sequences were kindly provided by Prof. Dr. Daniel Van Damme (VIB-UGhent Center for Plant Systems Biology, Gent, Belgium) [57] and plasmids containing G3BP-RFP sequences were kindly provided by the lab of Prof. Dr. Peter Tompa (VIB-VUB Structural Biology Research Center, Brussels, Belgium) [58]. All vectors were introduced into *Agrobacterium tumefaciens* (strains C58C1 PMP90 RifR, GV3101 or UIA143) by electroporation. Fully expanded leaves of 3-5 weeks old *N. benthamiana* were transiently transformed with an Agrobacterium strain carrying one of the fluorescent protein or double transformed transiently (for colocalization) with a 1:1 mixture of two *Agrobacterium* strains, each adjusted to OD600 of 0.4. Abaxial epidermal cells were analyzed microscopically, 2–4 days post-infiltration. Plasmolysis in *A. thaliana* root cells was achieved by incubation with 0.8 M sorbitol (VWR, Radnor, PA, United States). Heat treatment was achieved by incubating plants at 37 °C for 45 min. The autophagy response was induced by applying pressure on a cover slip just before the microscopy analysis.

### 4.3. Confocal Microscopy

Confocal images (16 bit) were acquired on a Zeiss LSM880 confocal microscope with Airyscan detector (Zeiss, Jena, Germany) or with a Nikon A1R confocal laser scanning microscope (Nikon Instruments) mounted on a Nikon Ti-E inverted epifluorescence body. A Plan-Apochromat 63×/1.4 oil objective was used to image in SR mode at a pixel size of 40 nm by 40 nm (Zeiss LSM880 confocal microscope) or an S plan Fluor ELWD 40× Ph2 ADM objective (NA 0.6) or CFI Plan Apo VC 60× WI DIC (NA 1.2) for the Nikon A1R confocal laser scanning microscope. EGFP was excited by the 488 nm laser line of an argon laser. A double band pass filter (BP 495-550) was placed in front of the Airyscan detector to detect EGFP emission (Zeiss LSM880 confocal microscope) or the emission filter was 515-530 nm for EGFP (Nikon A1R confocal laser scanning microscope). PI was excited with the 561 nm laser line of a diode laser. To detect PI emission, a combination of a BP 570-620 with a LP 645 filter was inserted in the light path. Images acquired with the Zeiss LSM880 confocal microscope were calculated through pixel reassignment and Wiener filtering by using the built-in ‘Airyscan Processing’ command in the Zen software. The images acquired with the Nikon A1R confocal laser scanning microscope were analyzed with Fiji software [59].

### 4.4. Recombinant ArathEULS3 Protein Production and Purification 

The construction of the vector carrying a HIS-tagged ArathEULS3 sequence was performed and described by Van Hove et al. (2011) [16]. *P. pastoris* strain X-33 was used for the expression of the recombinant ArathEULS3 protein as described by Al Atalah et al. (2011). The recombinant protein was purified from pelleted cells extracted in 20 mM 1,3-diaminopropane (pH 9, Sigma-Aldrich) in the presence of acid-washed glass beads (ø 250–500 μm). All supernatants were collected and the pH was set to 9 before loading on the Q Fast Flow column equilibrated in 20 mM 1,3-diaminopropane. After washing, the column was eluted with 0.1 M Tris-HCl pH 8.7 containing 0.5 M NaCl. The eluted samples were pooled and imidazole (Merck, Kenilworth, NJ, USA) was added to a final concentration of 25 mM before loading on a nickel NTA agarose column. After washing with washing buffer (0.1 M Tris-HCl, 0.5 M NaCl, and 25 mM imidazole, pH 8.7) a stepwise elution of the column was performed with 20 mM 1,3-diaminopropane pH 8.7 and 0.5 M NaCl, with increasing imidazole concentrations ranging from 50 to 250 mM imidazole. The eluted fractions were analyzed with SDS-PAGE and Western blot. Fractions considered to be pure were pooled, dialyzed against 20 mM 1,3-diaminopropane, and subsequently lyophilized and used for pull-down analysis.

### 4.5. SDS-PAGE and Western Blot Analysis

SDS-PAGE analysis was performed on 15% acrylamide gels. The proteins were visualized by gel staining with Coomassie Brilliant Blue R-250 or Pierce™ Silver Stain Kit (Thermo Scientific, Waltham, MA, United States). For Western blot analysis, samples separated by SDS-PAGE were electrotransferred to 0.45 µm polyvinylidene fluoride (PVDF) membranes (Fluorotrans^®^ PVDF, Pall Laboratory, Portsmouth, UK). After blocking the membranes in Tris-Buffered Saline (TBS: 10 mM Tris, 150 mM NaClm and 0.1% (*v/v*) Triton X-100, pH 7.6) containing 5% (*w/v*) non-fat milk powder (VWR), blots were incubated for 1 h with a rabbit polyclonal anti-EUL antibody, diluted 1/500 in TBS. The secondary antibody was a 1/1000 diluted goat anti-rabbit IgG labeled with horseradish peroxidase (Dako Cytomation, Glostrup, Denmark). Immunodetection was achieved by a colorimetric reaction using 700 µM 3,3′-diaminobenzidine tetrahydrochloride (Sigma-Aldrich) containing 0.003% (*v/v*) hydrogen peroxide dissolved in 0.1 M Tris-HCl buffer (pH 7.6).

### 4.6. Pull-down Analysis

In the pull-down assay a plant lysate from 2-week-old *Arabidopsis* plants subjected to 5 h of 100 µM ABA treatment was used as a prey. Wild type *A. thaliana* seeds, ecotype Columbia were germinated and grown in vitro on top of a 20 µM nylon mesh overlaid on ½ MS medium. Fourteen-day-old seedlings were subjected to 100 µM ABA treatment by transferring seedlings on the mesh to a new Petri dishes filled with ½ MS liquid medium containing the indicated concentration of ABA, incubated at 21 °C for 5 h, collected and frozen in liquid nitrogen. Since the stock solution for ABA was made in ethanol, control plants were kept on liquid medium containing 0.001% ethanol. Proteins were extracted in 25 mM Tris-HCl buffer containing 15 mM MgCl_2_, 150 mM NaCl, 0.1% NP-40, 1 mM PMSF and 1 µM E64, 0.1 % (*v/v*) benzonase, pH 7.6. Samples were vortexed 10 × 15 sec and incubated for 30 min at 4 °C on a rotating wheel. Finally, the protein extracts were centrifuged twice for 20 min at 13,000 rpm at 4 °C and the supernatant was collected and used as prey. ArathEULS3 protein dissolved in 100 mM Tris-HCl and 100 mM NaCl, pH 8.7 was used as a bait. 100 µg purified Myc-tagged ArathEULS3 protein was immobilized on 150 µL of Myc-Trap^®^ beads (Chromotek, Planegg, Germany), washed three times with 10 mM Tris-HCl, pH 7.5, 150 mM NaCl, and 0.5 mM EDTA and incubated for 30 min at 4 °C on a rotating wheel. From ABA-treated *A. thaliana* seedlings containing prey proteins, 300 µg of the protein lysate was added and incubated for 1 h at 4 °C on a rotating wheel before washing with 10 mM Tris-HCl, pH 7.5, 150 mM NaCl and 0.5 mM EDTA and three times with trypsin digest buffer (20 mM Tris-HCl, pH 8, and 2 mM CaCl_2_) and resuspended in 150 μL of the same buffer to store at −20 °C prior to MS analysis. All steps described above were performed on ice. Protein lysate from 2-week-old *Arabidopsis* plants subjected to 5 h of 100 µM ABA treatment used as prey incubated with empty Myc-Trap^®^ beads was used as a control. Four replicates for the control as well as for test pull-down experiment were performed. One replicate was analyzed by SDS-PAGE and silver staining and the remaining three replicates were analyzed by mass spectrometry. The samples were treated with 1 µg trypsin (Promega, Madison, WI, USA) for 4 h at 37 °C to cleave all proteins from the beads. After removal of the beads, the proteins were further digested with 1 µg trypsin overnight at 37 °C. The resulting peptide mixture was acidified by addition of 1 % trifluoroacetic acid (TFA). Next, peptides were purified on OMIX C18 tips (Agilent, Santa Clara, CA, United States). The tips were first washed 3 times with 150 µL pre-wash buffer (0.1% TFA in water/acetonitrile (ACN; 20:80, *v/v*)) and pre-equilibrated 5 times with 150 µL of solvent A (0.1% TFA in water/ACN (98:2, *v/v*)) before samples were loaded on the tip. After peptide binding, the tip was washed 3 times with 150 µL of solvent A and peptides were eluted twice with 150 µL elution buffer (0.1% TFA in water/ACN (40:60, *v/v*)).

### 4.7. LC−MS/MS Analysis

Purified peptides were dried, redissolved in solvent A and one-tenth of each sample was injected for LC–MS/MS analysis on an Ultimate 3000 RSLC nano LC (Thermo Fisher Scientific, Bremen, Germany) in-line connected to a Q Exactive mass spectrometer (Thermo Fisher Scientific) equipped with a pneu-Nimbus dual ion source (Phoenix S&T). Trapping was performed at 10 μL/min for 4 min in solvent A on a 10 mm µPACTM trapping column (PharmaFluidics, Ghent, Belgium,) with C18-endcapped stationary phase and the samples were loaded on a 50 cm long micro pillar array column (PharmaFluidics) with C18-endcapped functionality mounted in the Ultimate 3000′s column oven at 35 °C. For proper ionization, a fused silica PicoTip emitter (10 µm inner diameter) (New Objective, Woburn, MA, USA) was connected to the µPAC™ outlet union and a grounded connection was provided to this union. Peptides were eluted by a non-linear increase from 1% to 50% MS solvent B (0.1% FA in water/ACN (2:8, *v/v*)) over 99 min, first at a flow rate of 750 nL/min, then at 300 nL/min, followed by a 5-min wash reaching 99% MS solvent B and re-equilibration with MS solvent A (0.1% FA in water/ACN (2:8, *v/v*)). The mass spectrometer was operated in data-dependent, positive ionization mode, automatically switching between MS and MS/MS acquisition for the 5 most abundant peaks in a given MS spectrum. The source voltage was 2.5 kV, and the capillary temperature was 275 °C. One MS1 scan (m/z 400−2000, AGC target 3 × 10^6^ ions, maximum ion injection time 80 ms), acquired at a resolution of 70,000 (at 200 *m/z*), was followed by up to 5 tandem MS scans (resolution 17,500 at 200 *m/z*) of the most intense ions fulfilling predefined selection criteria (AGC target 5 × 10^4^ ions, maximum ion injection time 80 ms, isolation window 2 Da, fixed first mass 140 m/z, spectrum data type: centroid, underfill ratio 2%, intensity threshold 1.3 × 10^4^, exclusion of unassigned, 1, 5-8, >8 positively charged precursors, peptide match preferred, exclude isotopes on, dynamic exclusion time 12 s). The HCD collision energy was set to 25% normalized collision energy and the polydimethylcyclosiloxane background ion at 445.120025 Da was used for internal calibration (lock mass). Data analysis was performed with MaxQuant (version 1.6.3.4) using the Andromeda search engine with default search settings including a false discovery rate set at 1% on both the peptide and protein level. Spectra were searched against the *A. thaliana* proteins in the Uniprot database (database release version of August 2019 containing 39,369 protein sequences; www.uniprot.org) supplemented with the sequence of ArathEULS3. The mass tolerance for precursor and fragment ions was set to 4.5 and 20 ppm, respectively, during the main search. Enzyme specificity was set as C-terminal to arginine and lysine, also allowing cleavage at proline bonds with a maximum of two missed cleavages. Variable modifications were set to oxidation of methionine residues and acetylation of protein N-termini. Matching between runs was enabled with a matching time window of 1.5 min and an alignment time window of 20 min. Only proteins with at least one unique or razor peptide were retained leading to the identification of 424 proteins. Proteins were quantified by the MaxLFQ algorithm integrated in the MaxQuant software. A minimum ratio count of two unique or razor peptides was required for quantification. Further data analysis was performed with the Perseus software (version 1.6.2.1) after loading the protein groups file from MaxQuant. Reverse database hits were removed and replicate samples were grouped. Proteins with less than three valid values in at least one group were removed and missing values were imputed from a normal distribution around the detection limit leading to a list of 262 quantified proteins that was used for further data analysis. Then, a *t*-test was performed (FDR = 0.01 and s0 = 1) to compare samples and generate a volcano plot. As an alternative visualization, proteins that were significantly different in the *t*-test were shown in a heat map after non-supervised hierarchical clustering. The proteomics analyses were outsourced to the Proteomics Expertise Center (Center for Medical Biotechnology, VIB-UGhent, Gent, Belgium). The glycosylation sites in all secreted proteins identified as putative interaction partners for ArathEULS3 were analyzed using NetOGlyc 4.0 (http://www.cbs.dtu.dk/services/NetOGlyc/).

### 4.8. ArathEULS3 Sequence Analysis

A sequence of ArathEULS3 was obtained from UNIPROT id: Q954P1 and submitted to the following analysis: secondary structure prediction with PsiPred, [60], disorder profile with Disopred3 [61], function prediction with FFPred3 [62], protein domain fold recognition with pDomTHREADER [63], and conserved domain prediction with NCBI Conserved Domains [64] all present in the PSIPRED Workbench [65]. Molecular modeling was attempted with Modeller v. 9.22 [66] using the templates suggested by domain fold recognition.

## Figures and Tables

**Table 1:** Proteins significantly enriched after LC–MS/MS analysis.

Gene Locus	Full Name	MS Identifier	Score *
At2g39050	Ricin B-like lectin EULS3	EULS3	323.3
At3g23390	60S ribosomal protein L36a	RPL36AA	69.7
At4g22010	Pectin esterase-like protein SKU5 similar 4	sks4	39.8
At2g21660	Glycine-rich RNA-binding protein 7 AtGRP7	CCR2	37.5
At5g47870	DNA repair RAD52-like protein 2, chloroplastic	RAD52-2	26.3
At4g13940	Adenosyl homocysteinase	SAHH1	85.8
At2g06850	Xyloglucan endotransglucosylase /hydrolase	XTH4	26.8
At3g01500	Beta carbonic anhydrase 1	BCA1	40.3
At2g44210	F4I1.2 uncharacterized protein	At2g44210	19.2
At1g47128	Cysteine proteinase	RD21A	36.4
AtCg00790	50S ribosomal protein L16	rpl16	24.5
At1g07920/30/40	Elongation factor 1-alpha 1/2/3	A1/A2/A3	107.2
At4g23680	At4g23680	F9D16_150	12.4
At5g43940	S-(hydroxymethyl)glutathione dehydrogenase GSNORI	ADH2	33.1
At3g15190	30S ribosomal protein S20	RPS20	34.4
At3G04790	Probable ribose-5-phosphate isomerase 3	RPI3	41.0
At1G20620	Catalase-3	CAT3	170.6
At3g01780	Protein TPLATE	TPLATE	25.4
At2g20420	Succinate--CoA ligase [ADP-forming] subunit beta	At2g20420	323.3
At3g53870	40S ribosomal protein S3-2	RPS3B	66.0
At1g74970	30S ribosomal protein S9	RPS9	60.0
At1g78630	50S ribosomal protein L13	RPL13	107.9
At1G79850	30S ribosomal protein S17	RPS17	18.6
At1g07660	Histone H4	At1g07660	77.8
At4g35090	Catalase-2	CAT2	323.3
AtCG00820	30S ribosomal protein S19	rps19	69.7
At1g11860	Aminomethyltransferase,	GDCST	39.8
At1G07320	50S ribosomal protein L4	RPL4	37.5
At1G35680	50S ribosomal protein L21	RPL21	26.3
At3G09440	Heat shock 70 kDa protein 3	HSP70-3	85.8
At5g54770	Thiamine thiazole synthase	THI1	26.8

* The protein group score is the product of individual posterior error probabilities of the peptides of a protein group, and includes a factor to take into account the number of peptides per protein group. The protein group score is similar to the posterior error probabilities, in that it provides a measure of the certainty of protein identification.

## Supplementary Materials

Supplementary materials can be found at https://www.mdpi.com/1422-0067/21/5/1659/s1.

## Abbreviations

ABA	Abscisic Acid
EEA	*Euonymus europeaus* agglutinin
EGFP	enhanced Green Fluorescent Protein
EUL	*Euonymus europaeus* related lectins
EV	Extracellular Vesicle
EXPO	Exocyst-Positive Organelle
IDR	Intrinsically Disordered Region
LP	Leaderless Protein
MVB	Multivesicular Body
PB	Protein Body
PI	Propidium Iodide
