# Supplementary material for: The ArathEULS3 Lectin Ends up in Stress Granules and Can Follow an Unconventional Route for Secretion
Source: Int J Mol Sci. 2020 Feb 28;21(5):1659. doi: 10.3390/ijms21051659 (PMC7084908; doi:10.3390/ijms21051659)
Supplement: Supplementary file 1 [file ijms-21-01659-s001.zip › Supplementary data Dubiel.docx]

# Supplementary data


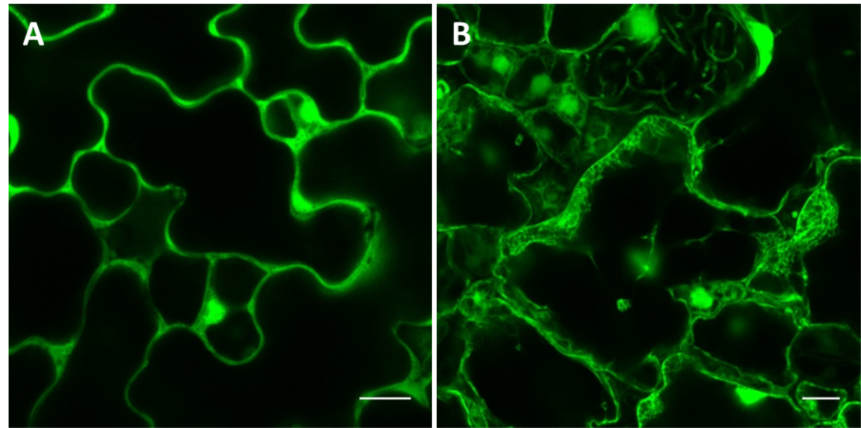


**Supplementary Figure S1.** Confocal images representing *A. thaliana* leaf epidermal cells stably expressing free EGPF before (A) and after 20 min of 1 M NaCl treatment (B). Images represent single slice optical sections. Scale bars represent 10 µm.


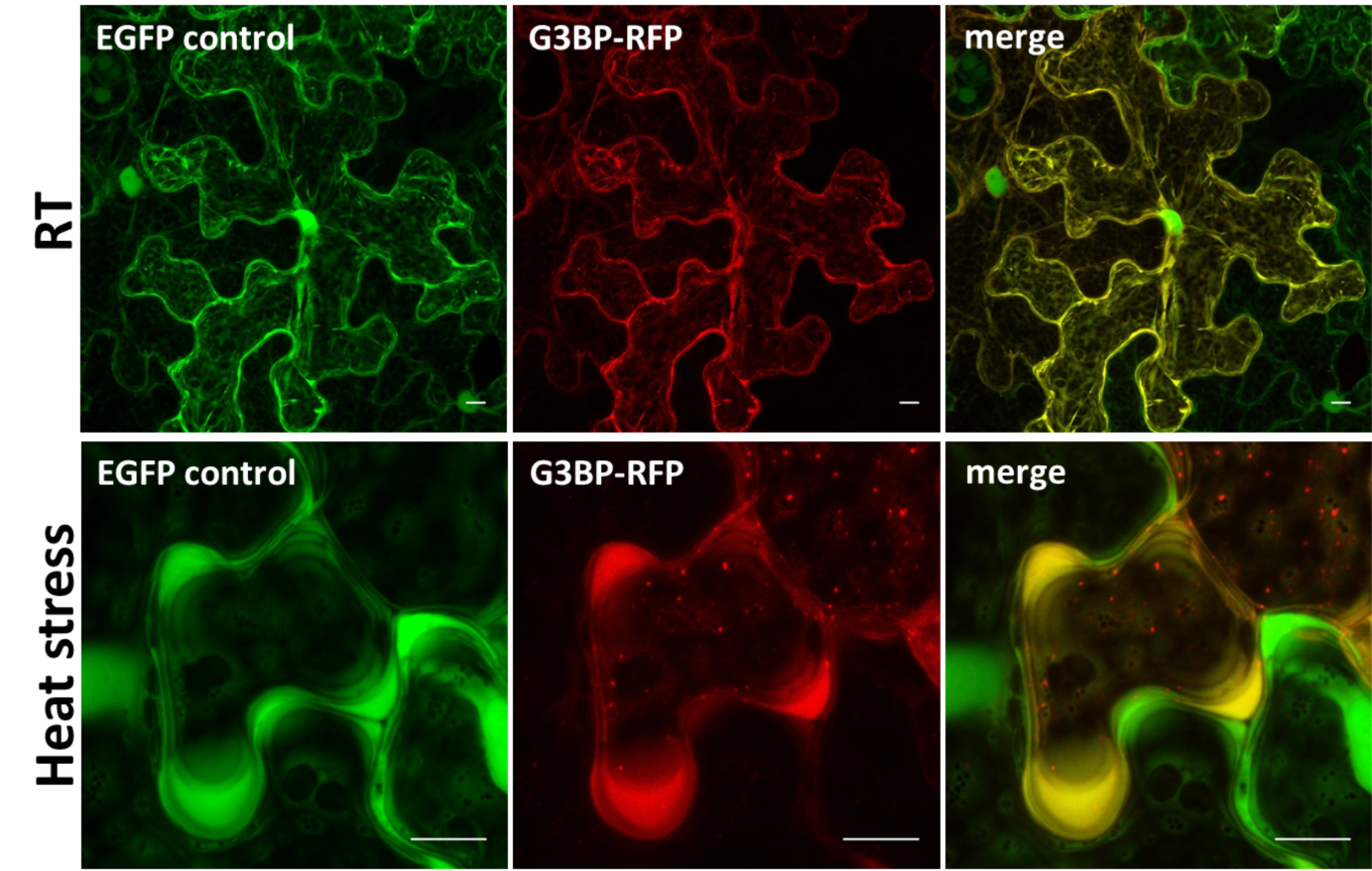


**Supplementary Figure S2.** Co-localization of free EGFP with G3BP-RFP stress granules marker. Upper images represent epidermal cells of *N. benthamiana* leaves after transient co-expression of free EGFP with G3BP-RFP at room temperature (RT) and lower images represent cells after 45 min of heat treatment at 37°C. Scale bars represent 10 µm.


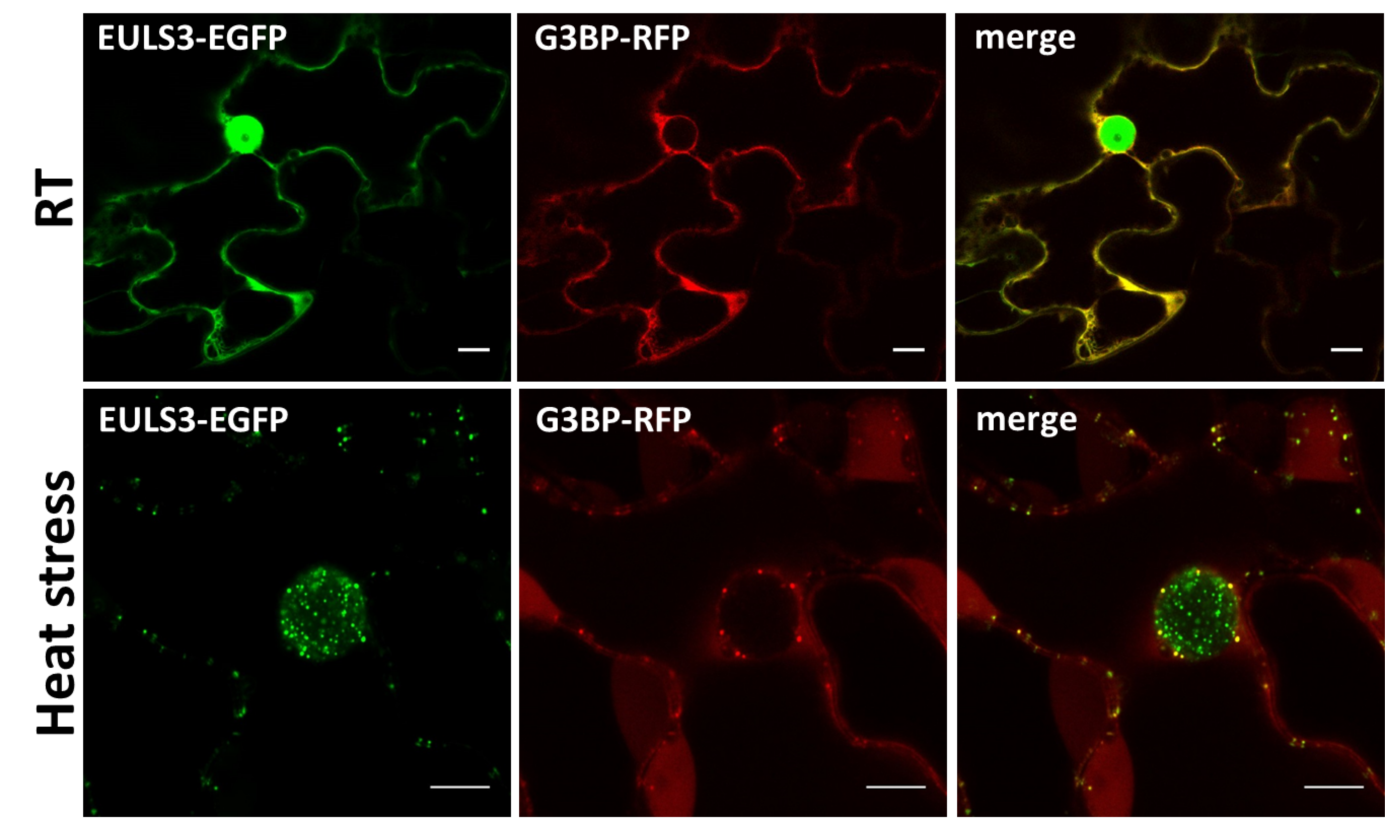


**Supplementary Figure S3.** Co-localization of ArathEULS3-EGFP with the G3BP-RFP stress granules marker. Upper confocal images represent epidermal cells of N*. benthamiana* leaves after transient co-expression of ArathEULS3-EGFP with G3BP-RFP at room temperature and lower images represent cells after 45 min of heat treatment at 37°C. All images represent a single slice optical section. Scale bars represent 10 µm.


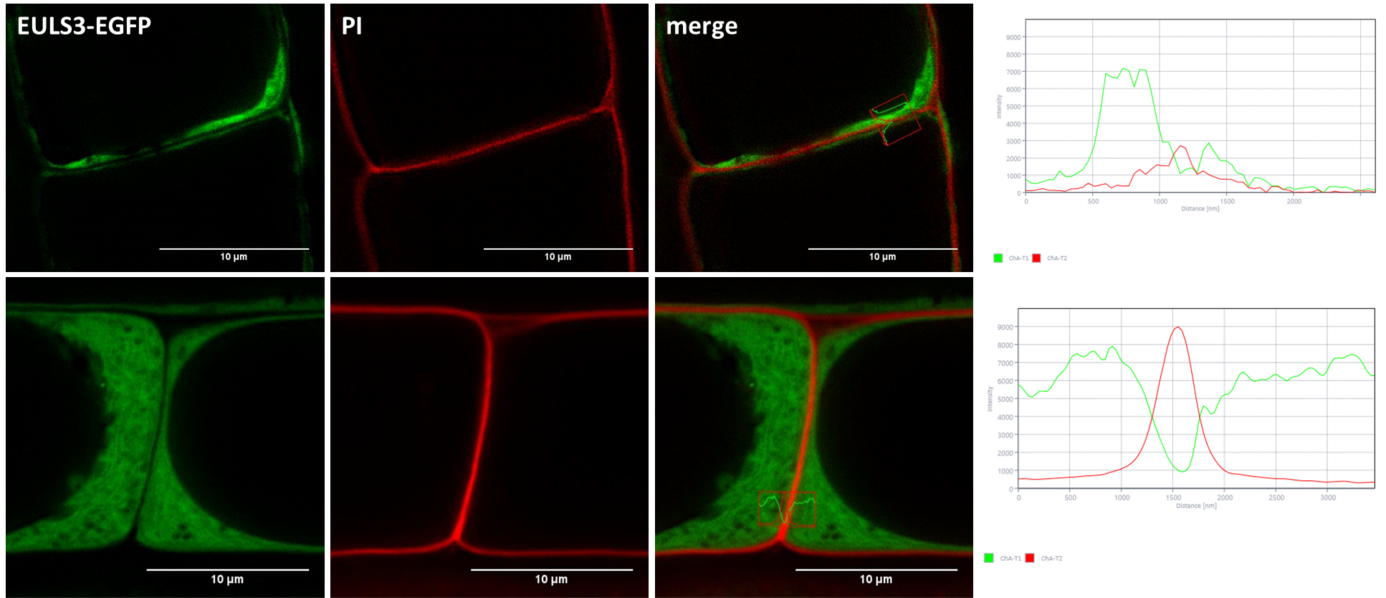


**Supplementary Figure S4. C**onfocal images representing *A. thaliana* root cells stably expressing ArathEULS3-EGFP. Cell walls were stained with PI. Co-localization of PI and EGFP staining was assessed in overlay pictures (merge). Scale bars represent 10 µm.


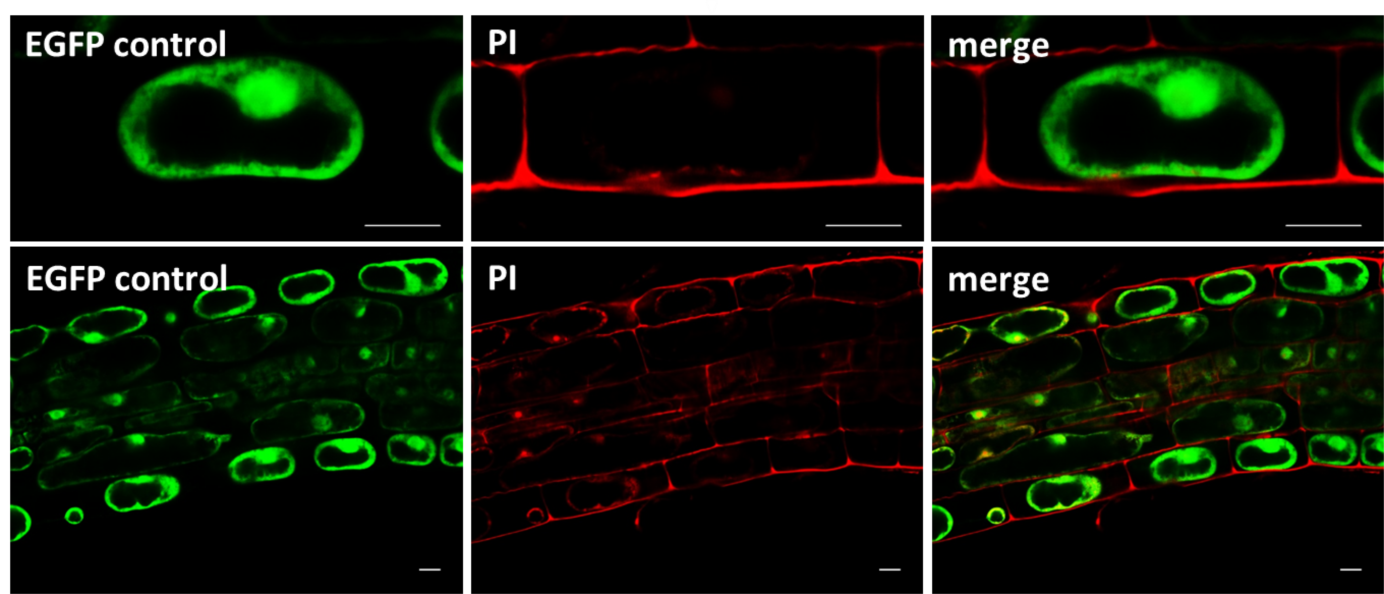


**Supplementary Figure S5.** Free EGFP localization in *A. thaliana* roots. Plasmolysis was performed using 0.8 M sorbitol. Confocal images show single slice optical section. Cell walls were stained with PI. Co-localization of PI and EGFP staining was assessed in overlay pictures (merge). Scale bars represent 10 µm.


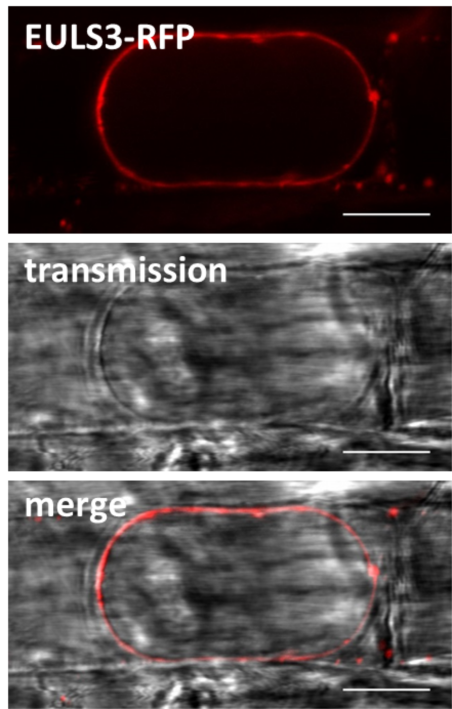


**Supplementary Figure S6.** ArathEULS3-RFP localization in *A. thaliana* roots. Confocal images showing fluorescence of ArathEULS3-RFP outside of the plant cell in plasmolyzed root cells treated with 0.8 M sorbitol. Confocal images show single slice optical sections. Cell walls were stained with PI. Co-localization of transmission and RFP signal was assessed in overlay pictures (merge). Scale bars represent 10 µm
